# Immuntherapie und Tyrosinkinaseinhibitoren beim metastasierten Nierenzellkarzinom in der First-line-Therapie – Wann welche Strategie?

**DOI:** 10.1007/s00120-020-01320-8

**Published:** 2020-10-07

**Authors:** G. Mickisch, I. Peters, C. Grüllich, T. Mudra, C. Doehn

**Affiliations:** 1 Centrum für Operative Urologie (COUB) Bremen, Robert-Koch-Str. 34a, 28277 Bremen, Deutschland; 2 Klinik für Urologie und Urologische Onkologie, Medizinische Hochschule Hannover, Hannover, Deutschland; 3 Klinik und Poliklinik für Urologie, Universitätsklinikum Dresden, Dresden, Deutschland; 4 APOGEPHA Dresden, Dresden, Deutschland; 5 Urologikum Lübeck, Lübeck, Deutschland

**Keywords:** Checkpointinhibitor, Sequenztherapie, Systemische Therapie, Kombination, Therapiewahl, Checkpoint inhibitors, Sequential treatment, Systemic treatment, Combination, Selection of therapy

## Abstract

Immuntherapien mit Checkpoint-Inhibitoren haben beim metastasierten klarzelligen Nierenzellkarzinom (mRCC) zu einem Paradigmenwechsel geführt und einen neuen Standard in der Erstlinie etabliert. Einschließlich der bekannten Monotherapie mit Tyrosinkinaseinhibitoren ist das Spektrum an medikamentösen Therapieoptionen somit breiter geworden. In diesem Beitrag sollen anhand der aktuellen Studiendaten sowie Leitlinienempfehlungen mögliche Faktoren zur individuellen Therapieplanung in der Erstlinie des mRCC diskutiert werden. Hierbei ist das wichtigste Leitkriterium das Risikoprofil. Daneben sind Effektivität und Verträglichkeit der Substanzen, sowie Tumorlast, Alter und Präferenzen der Patienten sowie Überlegungen zur Sequenztherapie für die Therapiewahl ausschlaggebend. Real-world-Daten für die neuen Kombinationstherapien, Biomarker für eine personalisierte Medizin sowie Studien zur optimalen Sequenztherapie beim mRCC werden benötigt.

## Abstract (en)

Immunotherapies with checkpoint inhibitors have led to a paradigm shift in metastatic renal cell carcinoma (mRCC) as they established a new standard in first-line treatment. In addition to the established monotherapy with tyrosine kinase inhibitors, the spectrum of first-line options has now become wider. Based on data from studies and current guideline recommendations, this article discusses possible factors for individual strategies in first-line treatment of mRCC. For this decision, the leading criterion is the patient’s risk score. In addition, the efficacy and tolerability of the substances, tumor burden, patient age and preferences as well as considerations about sequence treatment can support the decision. Real-world data for the new combination treatment, biomarkers for personalized medicine as well as studies on optimal sequence treatment for mRCC are needed.

## Einleitung

Immunonkologische Kombinationen aus zwei Checkpoint-Inhibitoren (CPI) und/oder aus CPI und Tyrosinkinaseinhibitoren (TKI) sind ein neuer Standard in der Erstlinientherapie des metastasierten Nierenzellkarzinoms (mRCC). In den jeweiligen Zulassungsstudien zeigten sich hohe Ansprechraten sowie eine damit verbundene lange Ansprechdauer [16–18]. Da die Ergebnisse der Studien aufgrund unterschiedlicher Studiendesigns und Patientenpopulationen nicht vergleichbar sind, bedarf es klinischer Kriterien, um für den individuellen Patienten die optimale Therapie auszuwählen.

## Status Quo in der Erstlinie: zugelassene Therapien

Die wichtigsten Therapien sind gegen den vaskulären endothelialen Wachstumsfaktor gerichtete Therapien (VEGFRi) und Immuntherapien mit CPI. Beide adressieren jeweils unterschiedliche Signalwege, die bei der Pathogenese des RCC eine Rolle spielen [8]. Eine Übersicht der CPI und TKI, die bei fortgeschrittenem und mRCC zugelassenen sind, findet sich in Tab. 1.

**Table 1:** 

Zugelassene Therapien mit CPI und TKI-Monotherapien		
Risikogruppe	Immuntherapeutische Kombinationen	TKI
Günstig	Pembrolizumab + Axitinib (PD-1-Inhibitor + TKI) Avelumab + Axitinib (PD-L1-Inhibitor + TKI)	Pazopanib Sunitinib Tivozanib Sorafenib^b^
Intermediär	Nivolumab + Ipilimumab (PD‑1 und CTLA4-Inhibitor) Pembrolizumab + Axitinib (PD-1-Inhibitor + TKI) Avelumab + Axitinib (PD-L1-Inhibitor + TKI)	Cabozantinib Pazopanib Sunitinib^a^ Tivozanib Sorafenib^b^
Ungünstig	Nivolumab + Ipilimumab (PD‑1 und CTLA-4-Inhibitor) Pembrolizumab + Axitinib (PD-1-Inhibitor + TKI) Avelumab + Axitinib (PD-L1-Inhibitor + TKI)	Cabozantinib Pazopanib Sunitinib^a^ Tivozanib Sorafenib^b^

*TKI* Tyrosinkinaseinhibitoren, *MSKCC* „Memorial Sloan Kettering Cancer“, *CPI* Checkpoint-Inhibitoren, *IMDC* „International Metastatic Renal Cell Carcinoma Database Consortium“, *PD-L1* „programmed cell death ligand 1“, *PD‑1* „programmed cell death 1“, *CTLA‑4* „cytotoxic t‑lymphocyte antigen 4“, *OS* Gesamtüberleben, *PFS* progressionsfreies Überleben, *ORR* Gesamtansprechrate

^a^In der CheckMate-214-Studie war Sunitinib bei Patienten mit intermediärer und ungünstiger Prognose gegenüber Nivolumab + Ipilimumab hinsichtlich der ORR und des OS unterlegen [17]

^b^Für Patienten, welche nicht für eine Interferon-α- oder Interleukin-2-basierte Therapie geeignet sind

Derzeit stehen Avelumab oder Pembrolizumab + Axitinib sowie Nivolumab + Ipilimumab als *immuntherapeutische Kombinationstherapien* zur Verfügung: Darüber hinaus sind die Kombination Bevacizumab + Interferon‑α sowie Temsirolimus (nur bei Hochrisikopatienten) zugelassen, sie spielen jedoch eine eher untergeordnete Rolle [8].

Neben den Zulassungsstatus ist die Stratifizierung in Risikogruppen gemäß IMDC-Score zur Therapieplanung von Bedeutung. Eine Übersicht über die einzelnen Kriterien findet sich in Tab. 2.

**Table 2:** 

IMDC-Risikofaktoren	Anzahl Risikofaktoren = Risikogruppe
Performancestatus <80 %	0 = günstig
Intervall von der Diagnose bis zur Systemtherapie <1 Jahr	
Hämoglobin unterhalb des Normwertes	1–2 = intermediär
Hyperkalzämie >10 mg/dl	
Neutrophile oberhalb des Normwertes	3–6 = ungünstig
Thrombozyten oberhalb des Normwertes	

*IMDC* „International Metastatic Renal Cell Carcinoma Database Consortium“

## Immuntherapien mit CPI: Überblick

Alle Kombinationstherapien mit CPI wurden in randomisierten, offenen Phase-III-Studien mit unterschiedlichen Patientenkollektiven im Vergleich mit Sunitinib geprüft. Bei den meisten Teilnehmern befand sich die Erkrankung im intermediären Stadium. Die Verteilung der Patienten über die Risikogruppen hinweg ist in den Studien uneinheitlich (Tab. 3). Auch die Raten an PD-L1-positiven Patienten waren unterschiedlich [16, 17, 20].

**Table 3:** 

	Nivolumab + Ipilimumab (*n* = 1096)	Pembrolizumab + Axitinib (*n* = 861)	Avelumab + Axitinib (*n* = 886)
	Daten für intermediäres und hohes Risiko gemäß IRRC	Daten für Gesamtkollektiv (alle Risikogruppen) gemäß BICR	Daten für PD-L1+ gemäß BICR
*Follow-up* *(Median in Monaten)*	49	23	9,9 (PFS) 11,6 (OS)
*Risikogruppen (%)*			
Günstig	23	32	22
Intermediär	61	55	64
Ungünstig	17	13	12
*PD-L1-Status (%)*			
≥1 %	23,0	59,5	63,2
*OS* *(Median in Monaten)*	**n.e.**	**n.e**	**n.e.**
HR (95 %-KI; *p*-Wert)	0,66 (0,55–0,80; <0,01)	0,68 (0,55–0,85; <0,0001)	0,82 (0,53–1,28; 0,38)
*PFS* *(Median in Monaten)*	**12,0**	**15,4**	**13,8**
HR (95 %-KI; *p*-Wert)	0,76 (0,63–0,91; <0,01)	0,71 (0,60–0,84; <0,001)	0,61 (0,47–0,79; <0,001)
*ORR (%)*	**42**	60,2	55,2
*p*-Wert	0,0001	<0,001	**–**
*Folgetherapien im Sunitinib-Arm (%)*	61	60,7	40
CPI	35^a^	37,6	24,1

Primäre Endpunkte sind fett markiert

*BICR* „blinded independent central review“, *IRRC* „independent radiology review committee“, *OS* Gesamtüberleben, *PFS* progressionsfreies Überleben, *ORR* Gesamtansprechrate, *PD-L1 *„programmed cell death ligand 1“, *PD‑1* „programmed cell death 1“, *CPI* Checkpoint-Inhibitor, *n.e.* nicht erreicht, *HR* Hazard Ratio, *KI* Konfidenzintervall

^a^Zweit- und weitere Therapielinien

Reife OS- und Langzeitdaten liegen bisher nur aus der CheckMate-214-Studie vor [23]. Eine Übersicht der Daten findet sich in Tab. 3.

### Nivolumab + Ipilimumab

In der CheckMate-214-Studie mit 1096 Teilnehmern betrugen die OS-Raten zum Zeitpunkt von 42 Monaten nach Randomisation bei Patienten mit intermediärem und hohem Risiko 52 % vs. 39 % zugunsten der Kombination (HR = 0,66; 95 %-KI: 0,55–0,80; *p* < 0,001). Auch das PFS (12,0 vs. 8,3 Monate; HR = 0,76; 95 %-KI: 0,63–0,91; *p* = 0,001) und die ORR fielen mit 42 % vs. 26 % (*p* < 0,001) und 10 % Komplettremissionen (CR) zugunsten von Nivolumab + Ipilimumab aus. Bei Patienten mit guter Prognose gab es einen statistisch signifikanten Vorteil bezüglich der Ansprechrate für Sunitinib. Nur 61 % der Patienten im Sunitinib-Studienarm erhielten eine Folgetherapie, nur bei 35 % war dies Nivolumab in der Zweitlinie [17, 23].

Unerwünschte Ereignisse (UE) von Grad 3–4 waren im CPI-Arm insgesamt seltener als unter Sunitinib (47 % vs. 64 %). Jedoch benötigten 35 % der Patienten im Nivolumab + Ipilimumab-Arm aufgrund immunvermittelter Nebenwirkungen eine Hochdosis Glukokortikoidtherapie. Jeder 5. Patient brach im Kombinationsarm die Behandlung aufgrund von Toxizitäten ab. Im Sunitinib-Arm waren UE länger anhaltend und 12 % der Patienten brachen die Therapie nebenwirkungsbedingt ab [17].

### Pembrolizumab + Axitinib

Die KEYNOTE-426-Studie umfasste 861 Patienten aller Risikogruppen. Zum Zeitpunkt von 24 Monaten nach Randomisation betrugen die OS-Raten 74 % vs. 66 % zugunsten der Kombination (HR = 0,68; 95 %-KI: 0,55–0,85; *p* < 0,001). Das mediane PFS lag bei 15,4 vs. 11,1 Monaten und die ORR betrug 60,2 % vs. 39,9 % (HR = 0,71; 95 %-KI: 0,60–0,84; *p* < 0,001). Der Nutzen der Kombination war unabhängig vom PD-L1-Status. In der Subgruppe mit günstigen Risikoprofil nach IMDC lagen die OS-Raten nach 24 Monaten bei 85 % im CPI-Arm und 88 % im Sunitinib-Arm (HR = 1,06; 95 %-KI: 0,60–1,86). Nur bei Patienten mit intermediären und ungünstigen Risikoprofil nach IMDC zeigte sich nach 24 Monaten ein statistisch signifikanter Vorteil im OS im CPI-Arm (HR = 0,63; 95 %-KI: 0,50–0,81). Im Sunitinib-Arm wurden 60,7 % der Patienten weiterbehandelt, davon 37,6 % mit einem CPI. Ähnlich wie in der CheckMate-214-Studie könnte der OS-Vorteil aufgrund dieser systematischen Untertherapie in der TKI-Kontrollgruppe überbewertet sein [18, 20].

Der Anteil therapiebedingter UE von Grad ≥3 betrug unter der CPI/TKI-Kombination 62,9 % im Vergleich zu 58,1 % im Sunitinib-Arm. In 30,5 % der Fälle führten UE jeder Ursache zum Absetzen einer der beiden Substanzen, bei 10,5 % zum Abbruch der kompletten Therapie und bei 69,9 % zu einer Therapieunterbrechung. Im Sunitinib-Arm waren UE bei 13,9 % der Patienten ursächlich für einen Therapieabbruch [20].

### Avelumab + Axitinib

Aus der JAVELIN-101-Studie mit 886 Patienten liegen vorläufige Daten mit einem Follow-up von 9,9 Monaten vor. Der Einschluss erfolgte unabhängig vom PD-L1-Status, jedoch waren die primären Endpunkte PFS und OS auf die PD-L1-positive (PD-L1+) Subgruppe ausgerichtet. Im Vergleich zur KEYNOTE-426-Studie handelte es sich um ein Kollektiv mit schlechteren prognostischen Kriterien (Tab. 3; [16]).

Mit einem medianen PFS von 13,8 vs. 7,2 Monaten zeigte sich im PD-L1+-Kollektiv eine signifikante Überlegenheit der Kombination (HR = 0,61; 95 %-KI: 0,47–0,79; *p* < 0,001). Dieser Vorteil war auch im Gesamtkollektiv zu sehen. Ein OS-Vorteil zeichnet sich derzeit nicht ab, finale Daten liegen noch nicht vor. Etwa 40 % der Patienten im Progress im Kontrollarm bekamen eine Zweitlinientherapie, davon 66,7 % einen CPI. Die Raten an UE von Grad ≥3 in beiden Armen waren vergleichbar [16].

## TKI: Überblick

Anti-VEGF(R)-gerichtete TKI-Therapien waren über mehr als 10 Jahre der dominierende Standard in der Erstlinienbehandlung des mRCC. Fast alle hierfür zugelassenen Wirkstoffe wurden in randomisierten, offenen Phase-III-Studien geprüft. Ausnahmen sind Pazopanib (doppelblindes Design) und Cabozantinib (Phase-II-Studie; [2, 14, 22]). In allen Studien waren die Patienten im Allgemeinstand nach „Eastern Cooperative Oncology Group“ (ECOG) ≤1. Lediglich in der CABOSUN-Studie befanden sich auch Patienten mit einem ECOG-Performancestatus 2. Zudem waren hier, ebenso wie in der Studie mit Tivozanib, auch Patienten mit ZNS-Metastasen vertreten [2, 15]. Der Fortschritt, der durch die Multikinasehemmer gegenüber der Zytokin-Ära erreicht wurde, zeigt sich in einem OS zwischen 26–43 Monaten [14, 19, 22].

### Sunitinib

Sunitinib war 2006 der erste beim mRCC zugelassene Multi-TKI. Das PFS verdoppelte sich in einem Kollektiv von 750 Patienten mit überwiegend günstigem und intermediärem Risiko mit dem TKI gegenüber Interferon‑α von 5 auf 11 Monate (HR = 0,42; 95 %-KI: 0,32–0,54; *p* < 0,001). Das mediane OS lag bei 26,4 und im Vergleichsarm bei 21,8 Monaten (HR = 0,82; 95 %-KI: 0,673–1,001; *p* = 0,051; [14]).

### Pazopanib

Pazopanib wurde vier Jahre später zugelassen. In einer Phase-III-Studie mit 425 Patienten, überwiegend mit günstigem und intermediärem Risiko, war der Wirkstoff in der Erstlinientherapie und nach vorangegangener Therapie mit Zytokinen hinsichtlich des PFS wirksamer als Placebo [22].

Die COMPARZ-Studie evaluierte die Effektivität von Sunitinib und Pazopanib im direkten Vergleich bei 1110 Patienten. Pazopanib war gegenüber Sunitinib in der unabhängigen Begutachtung bezüglich des PFS (8,4 vs. 9,5 Monate: HR = 1,05; 95 %-KI: 0,90–1,22) und des OS (28,3 vs. 29,1 Monate; HR = 1,05; 95 %-KI: 0,90–1,22) nicht unterlegen. Hinsichtlich der ORR erwies sich Pazopanib mit 31 % vs. 25 % im Vergleich mit Sunitinib als signifikant überlegen. Zudem sprachen die Patienten schneller auf Pazopanib an (11,9 vs. 17,4 Wochen; *p* = 0,03; [13]).

### Cabozantinib

Cabozantinib wurde in einer randomisierten Phase-II-Studie bei 157 Patienten mit intermediärem und ungünstigem Risiko evaluiert. Die Studie ergab einen signifikanten PFS-Vorteil gegenüber Sunitinib von 8,2 vs. 5,6 Monaten (HR = 0,66; 95 %-KI: 0,46–0,95; *p* = 0,012), wobei die Therapie mit Cabozantinib doppelt so lang dauerte wie im Vergleichsarm. Diese Daten aus der Analyse der Studienärzte bestätigten sich in einer unabhängigen Auswertung. Bezüglich des OS zeigte sich kein statistisch signifikanter Unterschied (26,6 vs. 21,2 Monate; HR = 0,80; 95 %-KI: 0,53–1,21; [2, 3]).

### Tivozanib

Tivozanib, ein sehr spezifischer VEGFR-Inhibitor, wurde in einer 2013 publizierten Studie bei mRCC-Patienten mit überwiegend günstigem und intermediärem Risikoprofil gegen den Zweitlinien-TKI Sorafenib geprüft (*n* = 517), aber erst 2017 – für alle Risikogruppen – zugelassen [1]. In der präspezifizierten Auswertung der Subgruppe der nicht vorbehandelten Patienten (*n* = 362) betrug das PFS 12,7 vs. 9,1 Monate (HR = 0,76; 95 %-KI: 0,58–0,99; *p* = 0,037). Ein OS-Vorteil wurde nicht gezeigt [15].

## Rationale für die Kombination von CPI mit TKI

**Figure 1:**
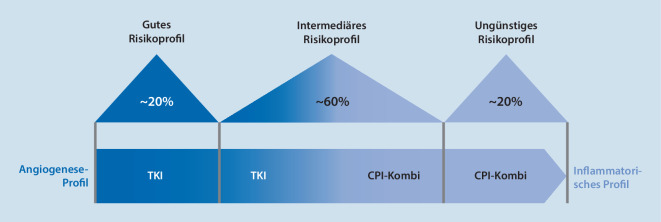

Die Tumorbiologie des RCC scheint bereits zu Beginn der Erkrankung hinsichtlich der Immunzellinfiltration sehr unterschiedlich zu sein. Darüber hinaus wird angenommen, dass sich die Tumorbiologie im Erkrankungsverlauf verändern kann: Eine zunehmende Inflammation wird hierbei diskutiert, wobei sich ein nicht immunogener „kalter“ Tumor zu einem immunogenen „heißen“ Tumor entwickeln könnte (Abb. 1). Eine Angiogenese sowie eine inflammatorische Achse unterschiedlicher Ausprägung wird beim RCC diskutiert [17, 20, 26, 27].

Unabhängig davon wird der Tumor durch eine TKI-Therapie sensibler für eine immunzellvermittelte Tumorlyse [7].

**Figure 2:**
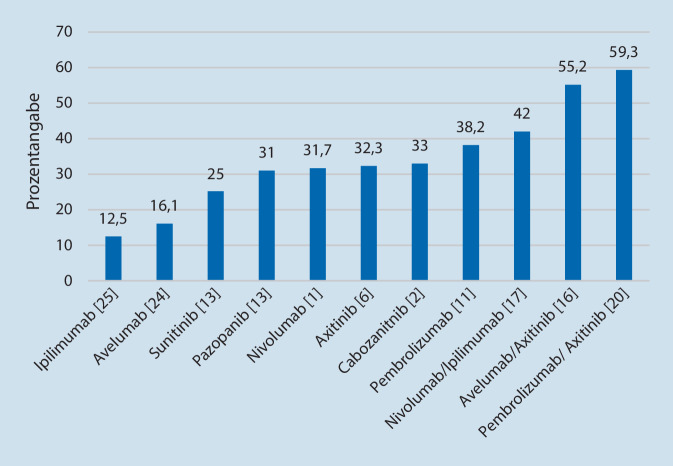

Diese theoretischen Erkenntnisse könnten erklären, warum die Kombination von CPI und TKI bei intermediärem Risiko besonders gut wirkt und die duale Immuntherapie aus zwei CPI in der CheckMate-214-Studie bei günstiger Prognose weniger wirksam war [17, 23]. Die bisher vorliegende Evidenz zeigt auch, dass hohe Ansprechraten beim mRCC erst durch die Kombination von CPI mit TKI erreicht werden (Abb. 2).

## Mögliche Entscheidungskriterien in der klinischen Praxis

Die Therapiewahl in der Erstliniensituation ist essenziell, da nur etwa 50 % der Patienten mit mRCC eine Zweitlinientherapie bekommen [10]. Nachfolgend werden relevante klinische Kriterien erläutert, die für die Therapieentscheidung eine Rolle spielen können. Eine Zusammenfassung findet sich in Tab. 4.

**Table 4:** 

Mögliche Faktoren für die Therapieentscheidung	IO-IO-Kombi	IO-TKI-Kombi	TKI-Mono
*Risikoscore*			
Günstig	–	+ (v. a. Pembrolizumab + Axitinib)	+ (Pazopanib/Sunitinib)
Intermediär low	+	+	+
Intermediär high	+	+	+/−
Ungünstig	+	+ (v. a. Avelumab + Axitinib)	+ (Cabozantinib)
*Hohe PD-L1-Expression*	+	+	–
*Schlechter Allgemeinzustand*	–	+/−	+
*Alter >75*	–	+/−	+
*Hohe Tumorlast/Symptomatik*	+	+ (v. a. Avelumab + Axitinib)	+ (v. a. Cabozantinib)
*Verträglichkeit*	–	+ Avelumab + Axitinib > Pembrolizumab + Axitinib	+ Pazopanib > Sunitinib 4/2
*Komorbiditäten*			
Kardiovaskuläre Ereignisse	+	−/+	–
Eingeschränkte Leberfunktion	–	–	+/− (Tivozanib, Sunitinib)
Autoimmunerkrankungen, Immunsuppression oder Kortikosteroidtherapie ≥10 mg/Tag	–	–	+
*Aufwand (Applikation Praxisausstattung, Aufklärung, Management)*	–	−/+	+
*Kosten*	–	−/+	+

+ Spricht eher dafür, +/− Neutral, − Spricht eher dagegen

*PD-L1 *„programmed cell death ligand 1“, *TKI* Tyrosinkinaseinhibitor

### Biomarker

Weder für die zielgerichtete noch für die Erstlinientherapie mit CPI gibt es derzeit beim mRCC validierte Biomarker. Alle drei Immuntherapiekombinationen sind unabhängig vom PD-L1-Status zugelassen, wobei in den Studien CheckMate-214 und KEYNOTE-426 PD-L1+-Patienten stärker profitieren als diejenigen ohne dieses Merkmal [17, 20]. In der JAVELIN-101-Studie war der PD-L1-Status offenbar prädiktiv für die Wirksamkeit von Sunitinib, aber nicht für die Immuntherapiekombination [16].

### Risikoscore

Der Risikoscore ist im Erstliniensetting zulassungsrelevant: Die Kombination Nivolumab + Ipilimumab war bei intermediärem und ungünstigem Risiko wirksamer als Sunitinib, bei günstiger Prognose jedoch nicht [17]. Von der Kombination von Pembrolizumab + Axitinib profitierten alle Risikogruppen, jedoch war der Unterschied im PFS bei Patienten mit günstigem Risiko vs. Sunitinib nicht signifikant (HR = 0,64; 95 %-KI: 0,24–1,68). Cabozantinib wurde nur bei intermediärer und ungünstiger Prognose geprüft und zugelassen [2, 20].

In allen CPI-Studien erfolgte die Stratifizierung gemäß IMDC-Score, in der JAVELIN-101-Studie auch nach den Kriterien des Memorial Sloan Kettering Cancer Center (MSKCC). Dieser sog. Motzer-Score war auch in allen TKI-Studien Standard. Die in der TKI-Ära zusammengefassten günstigen und intermediären Prognosegruppen müssen auf Basis der Studiendaten zu den Immuntherapien wieder separat betrachtet werden, wobei das am häufigsten vorliegende intermediäre Risikoprofil sehr heterogen ist [14–17, 20, 22].

### Tumor- und Patientencharakteristika

Auf Basis der Studiendaten kommen bei intermediärem und ungünstigem Risiko sowie bei hoher Tumorlast, ausgeprägter Symptomatik und jüngeren Patienten in gutem Allgemeinzustand die Kombinationen Nivolumab + Ipilimumab, Pembrolizumab + Axitinib, Cabozantinib sowie Avelumab + Axitinib infrage. Zu berücksichtigen ist, dass für Nivolumab + Ipilimumab sowie Pembrolizumab + Axitinib für Patienten über 75 Jahren keine gesicherten Daten vorliegen, während die TKI Sunitinib und Pazopanib unabhängig vom Alter wirksam sind [16–18, 20, 21, 23].

### Verträglichkeit

Da die CPI-haltigen Therapien noch nicht lange zugelassen sind, sind praktische Erfahrungen zum Therapiemanagement limitiert. Die Toxizität ist höher als bei Monotherapien mit CPI und ist der wichtigste Grund für Therapieabbrüche. Insbesondere immunassoziierte Nebenwirkungen (irAE) sind ein neues Feld und gehen mit anderen Anforderungen bezüglich des Managements und Monitorings einher. Die EMA hat ausdrücklich Schulungs- und Informationsprogramme gefordert.

Die IrAE können verschiedene Organsysteme betreffen. Am häufigsten treten Hautprobleme, endokrine Nebenwirkungen, Kolitis, Diarrhö, Hepatitis und Pneumonitis auf, auch infusionsbedingte Reaktionen sind relevant. In der Praxis kann es schwierig sein, Toxizitäten den ursächlichen Substanzen zuzuordnen und entsprechend zu handhaben.

Die Behandlung von irAE erfolgt überwiegend mit Kortikosteroiden, in schweren Fällen auch mit Immunsuppressiva. In Notfallsituationen muss ein schneller Zugang zu einer multidisziplinären Therapie gewährleistet sein, dies gilt vor allem für die duale CPI-Therapie. Hier gab es doppelt so viele Therapieabbrüche wie unter Sunitinib [17, 23]. In der KEYNOTE-426-Studie war bei fast zwei Dritteln der Patienten eine Therapiepause notwendig, zudem wurde etwa ein Drittel nebenwirkungsbedingt mit einer Monotherapie weiterbehandelt [20].

Das Nebenwirkungsprofil von VEGF(R)-gerichteten TKI ist bekannt und das Management mittlerweile Routine. Zu den klassentypischen Toxizitäten gehören Bluthochdruck, Rash, Fatigue, Hand-Fuß-Syndrom, Diarrhö, Mukositis, kardiale Nebenwirkungen und Leberwerterhöhungen. In der PISCES-Studie war Pazopanib besser verträglich als Sunitinib und wurde von den Patienten daher präferiert [4, 19, 21].

### Applikation der Therapie

Die CPI werden als Infusion in verschiedenen Intervallen gegeben: Nivolumab + Ipilimumab alle 3 Wochen (4 Zyklen lang, danach Erhaltung mit Nivolumab) sowie Pembrolizumab + Axitinib alle 3 Wochen, Avelumab + Axitinib alle 2 Wochen. Axitinib wird oral 2‑mal täglich eingenommen [16, 17, 20]. Immuntherapien werden besser vergütet als TKI, allerdings muss die Klinik oder Praxis entsprechend ausgestattet sein und über qualifiziertes Personal verfügen. Daher ist zu erwarten, dass sich die Therapie stärker von der allgemeinurologischen Praxis in Richtung spezialisierter urologischer oder internistischer Onkologie mit geeigneter Infrastruktur verlagert. TKI sind aufgrund der oralen Einnahme für den Patienten komfortabler, jedoch ist die Compliance schwer zu überprüfen. Das Nebenwirkungsmanagement von Kombinationstherapien mit CPI könnte im niedergelassenen Bereich eine Herausforderung darstellen.

### Erfahrung

Für TKI liegen langjährige Erfahrungen aus der klinischen Praxis und Real-world-Daten vor. So erreichten Studien-ungeeignete mRCC-Patienten in der ADONIS-Studie mit Sunitinib und in der PRINCIPAL-Studie mit Pazopanib ein OS von fast 34 Monaten [19, 21].

### Patientenpräferenz

Da es sich um eine palliative Therapie handelt, stehen hier neben der Wirksamkeit v. a. die Verträglichkeit und die Lebensqualität im Fokus. Dies bestätigte sich in der PISCES-Studie [4]. Auch Komorbiditäten und die individuelle Lebenssituation spielen eine Rolle. Diese hat möglicherweise auch Einfluss auf die Art der Applikation, die im Falle einer Immuntherapie Mobilität voraussetzt. Damit Patienten in die Lage versetzt werden, partizipativ mitentscheiden zu können, müssen sie gut aufgeklärt werden. Für ein solches Gespräch können 30 bis 45 min eingeplant werden.

### Empfehlungen in Leitlinien

Die Empfehlungen der Leitlinien sind derzeit nicht einheitlich. Die EAU-Guideline empfiehlt die Kombination von Avelumab mit Axitinib aufgrund des ausstehenden Nachweises eines OS-Vorteils derzeit nicht [9, 16]. Eine Übersicht befindet sich in Tab. 5.

**Table 5:** 

	Prognosegruppe (IMDC)		
	Günstig	Intermediär	Ungünstig
*EAU*			
Standard	Pembrolizumab + Axitinib	Nivolumab + Ipilimumab Pembrolizumab + Axitinib	Nivolumab + Ipilimumab Pembrolizumab + Axitinib
Optional	Pazopanib Sunitinib	Pazopanib Cabozantinib Sunitinib	Pazopanib Cabozantinib Sunitinib
*ESMO*			
Standard	Pembrolizumab + Axitinib	Pembrolizumab + Axitinib Nivolumab + Ipilimumab	Pembrolizumab + Axitinib Nivolumab + Ipilimumab
Optional	Pazopanib Sunitinib Tivozanib	Pazopanib Sunitinib Cabozantinib	Pazopanib Sunitinib Cabozantinib
*S3-Leitlinie*			
Standard	Pembrolizumab + Axitinib Avelumab + Axitinib*	Nivolumab + Ipilimumab Pembrolizumab + Axitinib Avelumab + Axitinib*	Nivolumab + Ipilimumab Pembrolizumab + Axitinib Avelumab + Axitinib*
Optional	Bevacizumab + IFN Pazopanib Sunitinib Tivozanib	Cabozantinib Sunitinib Pazopanib Tivozanib Bevazizumab + IFN	Cabozantinib Sunitinib Pazopanib Temsirolimus

*EAU* European Association of Urology, *ESMO* European Society for Medical Oncology, *IMDC* „International Metastatic Renal Cell Carcinoma Database Consortium“

* Zu dieser Kombination liegen noch keine hinreichenden Überlebensdaten vor

### Sequenztherapie

Überlegungen zur Sequenztherapie müssen in die Erstlinienentscheidung einfließen. Bereits in der TKI-Ära gab es hierzu kontroverse Diskussionen, die auf dem Boden der Immuntherapie gewissermaßen neu startet, weil es keinerlei verlässliche Evidenz für Zweitlinientherapie nach einer immunonkologischen Kombinationstherapie gibt.

Daten zur Zweitlinientherapie nach einer TKI-Monotherapie liegen für Nivolumab, Cabozantinib, Lenvatinib + Everolimus und Axitinib (nach Sunitinib) vor [9, 12, 21]. Nivolumab hat auf Basis einer gesicherten Evidenz einen höheren Stellenwert, da hiermit ein Wechsel des Wirkmechanismus stattfindet und es sich um eine gut verträgliche Therapie handelt [12]. Zudem ist damit gewährleistet, dass die Patienten im Therapieverlauf einen CPI bekommen. Ein hoher Remissionsdruck würde dagegen eher für Cabozantinib sprechen [2, 3].

Da in den TKI-Kontrollarmen der CheckMate-214- und KEYNOTE-426-Studie in eher geringem Maße effektive Folgetherapien eingesetzt wurden, bleibt unklar, ob eine geeignete Sequenztherapie nach einer TKI-Monotherapie in der Erstlinie nicht zu vergleichbaren Ergebnissen bei möglicherweise besserer Verträglichkeit gekommen wäre. Denkbar ist daher auch ein anderer Ansatz. Da die Erhöhung der abnormalen Gefäßdichte und Dysregulation von Immunzellen den immunsuppressiven Effekt der Tumorumgebung verstärken, scheint die antiangiogene Therapie biologisch gesehen sinnvoll, um die spätere Aktivität einer Immuntherapie zu erhöhen [16, 17, 20].

## Fazit für die Praxis

- Die Erstlinientherapie des metastasierten klarzelligen Nierenzellkarzinoms (mRCC) richtet sich primär nach dem IMDC-Risikoscore („International Metastatic Renal Cell Carcinoma Database Consortium“).
- In die interdisziplinäre Diskussion über die Therapiewahl müssen Wirksamkeit, Verträglichkeit, Nebenwirkungsprofil und Überlegungen zur Sequenztherapie einfließen.
- Immunonkologische Therapien sollten möglichst von erfahrenen Praxen oder Kliniken durchgeführt werden.
- Bei intermediärem und ungünstigem Risiko sowie hohem Remissionsdruck bieten sich eine CPI/TKI- (Checkpoint-Inhibitoren/Tyrosinkinaseinhibitoren-) oder CPI/CPI-Kombination sowie Cabozantinib an.
- TKI der ersten Generation haben nach wie vor ihre therapeutische Berechtigung bei günstigem Risikoprofil, Kontraindikationen für eine Immuntherapie, langsamem Erkrankungsverlauf, reduziertem Allgemeinzustand und hohem Alter.
- Derzeit gibt es keine Evidenz für eine Zweitlinientherapie nach Versagen einer CPI-haltigen Vortherapie. Die initiale Therapie mit einem TKI lässt die Möglichkeit eines CPI-Einsatzes in der Zweitlinie offen.
- Es werden validierte Biomarker benötigt.
