# Erratum zu: Immuntherapie und Tyrosinkinaseinhibitoren beim metastasierten Nierenzellkarzinom in der First-line-Therapie – Wann welche Strategie?

**DOI:** 10.1007/s00120-021-01478-9

**Published:** 2021-03-03

**Authors:** G. Mickisch, I. Peters, C. Grüllich, T. Mudra, C. Doehn

**Affiliations:** 1 Centrum für Operative Urologie (COUB) Bremen, Robert-Koch-Str. 34a, 28277 Bremen, Deutschland; 2 Klinik für Urologie und Urologische Onkologie, Medizinische Hochschule Hannover, Hannover, Deutschland; 3 Klinik und Poliklinik für Urologie, Universitätsklinikum Dresden, Dresden, Deutschland; 4 APOGEPHA Dresden, Dresden, Deutschland; 5 Urologikum Lübeck, Lübeck, Deutschland

**Erratum zu:**

**Urologe 2021**

10.1007/s00120-020-01320-8

Der Artikel „Immuntherapie und Tyrosinkinaseinhibitoren beim metastasierten Nierenzellkarzinom in der First-line-Therapie – Wann welche Strategie?“ von G. Mickisch, I. Peters, C. Grüllich, T. Mudra und C. Doehn wurde ursprünglich Online First ohne „Open Access“ auf der Internetplattform des Verlags publiziert. Nach der Veröffentlichung in Band 59, Heft 12, pp. 1504–1511 hatten sich die Autoren für eine „Open Access“-Veröffentlichung entschieden. Das Urheberrecht des Artikels wurde deshalb in © Der/die Autor(en) 2020 geändert.

